# Cortical Network Topology in Prodromal and Mild Dementia Due to Alzheimer’s Disease: Graph Theory Applied to Resting State EEG

**DOI:** 10.1007/s10548-018-0674-3

**Published:** 2018-08-25

**Authors:** Raffaella Franciotti, Nicola Walter Falasca, Dario Arnaldi, Francesco Famà, Claudio Babiloni, Marco Onofrj, Flavio Mariano Nobili, Laura Bonanni

**Affiliations:** 1 Department of Neuroscience, Imaging and Clinical Science, “G. d’Annunzio” University, Via Luigi Polacchi, 66013 Chieti, Italy; 2 BIND – Behavioral Imaging and Neural Dynamics Center, “G. d’Annunzio” University, Chieti, Italy; 3 Dipartimento di Neuroscienze (DINOGMI), Università di Genova, Genoa, Italy; 4 U.O. Clinica Neurologica, IRCCS Ospedale Policlinico San Martino, Genoa, Italy; 5 U.O. Neurofisiopatologia, IRCCS Ospedale Policlinico San Martino, Genoa, Italy; 6 Department of Physiology and Pharmacology, University of Rome “La Sapienza”, Rome, Italy; 7 IRCCS S. Raffaele Pisana, Rome, Italy; 8 IRCCS S. Raffaele Cassino, Cassino, Italy

**Keywords:** Granger causality, Graph theory, Integration, Mild cognitive impairment, Resilience, Segregation

## Abstract

Graph theory analysis on resting state electroencephalographic rhythms disclosed topological properties of cerebral network. In Alzheimer’s disease (AD) patients, this approach showed mixed results. Granger causality matrices were used as input to the graph theory allowing to estimate the strength and the direction of information transfer between electrode pairs. The number of edges (degree), the number of inward edges (in-degree), of outgoing edges (out-degree) were statistically compared among healthy controls, patients with mild cognitive impairment due to AD (AD-MCI) and AD patients with mild dementia (ADD) to evaluate if degree abnormality could involve low and/or high degree vertices, the so called hubs, in both prodromal and over dementia stage. Clustering coefficient and local efficiency were evaluated as measures of network segregation, path length and global efficiency as measures of integration, the assortativity coefficient as a measure of resilience. Degree, in-degree and out-degree values were lower in AD-MCI and ADD than the control group for non-hubs and hubs vertices. The number of edges was preserved for frontal electrodes, where patients’ groups showed an additional hub in F3. Clustering coefficient was lower in ADD compared with AD-MCI in the right occipital electrode, and it was positively correlated with mini mental state examination. Local and global efficiency values were lower in patients’ than control groups. Our results show that the topology of the network is altered in AD patients also in its prodromal stage, begins with the reduction of the number of edges and the loss of the local and global efficiency.

## Introduction

Alzheimer’s disease (AD) is the most common form of dementia (Reitz et al. 2011). AD dementia is preceded by a preclinical period characterized by the absence of overt symptoms (Price et al. 2009). It is supposed that in that period, the process progresses until it crosses a threshold to clinically recognizable dysfunction (Thal et al. 2002; Braak et al. 2011). According to the most recent guidelines (McKhann et al. 2011; Dubois et al. 2014), AD can be diagnosed before the appearance of any cognitive or behavioral symptoms, i.e., in a preclinical (before any objective cognitive deficit) or prodromal (mild cognitive impairment, AD-MCI) stage (Petersen et al. 2009), based on pathophysiological diagnostic markers revealed by CSF and positron emission tomography (PET) biomarkers of Aβ-1-42 and tau in the brain. However, these guidelines encourage the research to develop other techniques that can enrich the non-invasive and inexpensive instrumental assessment, including topographic biomarkers of preclinical and prodromal stages of AD. The topographic biomarkers include brain hypometabolism, as revealed by ^18^F-fluorodeoxyglucose PET (FDG-PET) and maps of brain atrophy and abnormalities of structural brain connectivity, as revealed by magnetic resonance imaging (MRI; Dubois et al. 2014).

Electroencephalography (EEG) has been proficiently applied to the study of dementia for long time, specifically in AD (van Straaten et al. 2014), showing a shift of the power spectrum to lower frequencies (Jeong 2004; Babiloni et al. 2006) and a decrease in coherence of fast rhythms (Locatelli et al. 1998; Jelles et al. 2008) in AD patients with dementia (ADD). In parallel to the computation of EEG power density, the analysis of functional connectivity on EEG data seems a promising method to provide additional topographic biomarkers of AD. The “functional brain connectivity” reflects a statistical dependence of a given variable linking the activity in different cerebral regions (Friston 2011). Furthermore, “effective connectivity” probes another dimension of functional connectivity referring to a causal influence from one to another neural region; it can be measured by Granger causality (GC; Blinowska and Zygierewicz 2011; Kaminski and Blinowska 2014; Seth et al. 2015). Together with conventional methods of EEG frequency and functional connectivity analysis, graph theory analysis provides a method to integrate the topology of the pair-wise functional connectivity values into one characterized network. A given network is defined by a collection of vertices and edges between pairs of vertices. Networks can be organized according to different models from regular lattices and trees to random networks. Regular graphs are organized in vertices which have the same number of edges. Highly connected vertices represent hubs which form tightly interconnected communities, the so-called rich clubs. Topological features include clustering coefficient, probing the tendency of network elements to form local clusters, cliques, or small groups of closely interconnected vertices, and the individual interconnection path length, defined as the length of the shortest paths connecting pairs of vertices, which quantifies the efficiency of information transmission within the network. Regular graphs tend to have long average path length and high clustering coefficient, whereas in random networks most vertices have the same number of edges, the average paths are short and the clustering coefficient is low. Other possible organization models are scale-free, small-world, modular, and hierarchical archetypes. Among these models, the small-world networks have an optimal balance between local specialization and global integration with similar path length but higher clustering than a random network (Watts and Strogatz 1998), making the network resilient to damage.

In the case of EEG, the vertices of a brain network are usually represented by the cerebral regions located under the electrodes, and the functional connectivity value of each electrode pair (represented in a connectivity matrix) is used as a functional connection among vertices. That value is typically computed with EEG quantitative measures of functional connectivity, such as coherence, phase lag index or synchronization likelihood, less frequently with effective connectivity. Functional connectivity measures evaluate the connectivity strength between pairs of vertices giving no information about the direction of the edge. In addition to the most commonly used unidirectional measures, effective connectivity measures not only the connectivity strength but also its direction.

Graph theory analysis of cerebral networks has been implemented in brain disease including AD (Stam 2014), challenging the classical concept of neurological disorders being either ‘local’ or ‘global’. Results of this analysis have pointed to the overload and failure of hubs as a possible final common pathway in neurodegenerative disorders. However, previous EEG studies on the comparison between AD patients and control subjects reported divergent results (de Haan et al. 2009; Tijms et al. 2013; Miraglia et al. 2016). Nevertheless, the results were inconsistent in some respect. It has been described either a longer characteristic path length together with a preserved clustering coefficient (Stam et al. 2007) or a shorter characteristic path length with a decreased clustering coefficient in AD patients compared to control subjects (de Haan et al. 2009). More research is therefore needed to determine the most consistent topographic pattern and mathematical measure to model abnormal topology of the functional coupling in AD in the prodromal (AD-MCI) compared to the dementia (ADD) stage (Dauwels et al. 2010a). Specifically, in the context of AD research, a methodological aspect not yet explored in the above EEG studies is the use of a mathematical approach which employs both the raw EEG signal in the time domain and the direction of the information transfer between vertices as an input to the graph theory analysis. The measure of effective connectivity in the time domain allows to evaluate all possible information transfers and to have a complete picture of the network organization with distinction between driver (i.e., a source vertex with zero incoming edges) and recipient (i.e., a sink vertex with zero outgoing edges), between hubs with main outgoing edges (broadcasters) and hubs with prevailing incoming edges (integrators).

In this exploratory study, we used GC analysis (Seth 2010; Franciotti et al. 2013; Falasca et al. 2015) to produce connectivity matrices as inputs to the graph theory analysis. These connectivity matrices include information on both the strength and the direction of the edges, so they are called weighted directed matrices. By means of graph theory analysis on weighted directed matrices we aim to test the hypothesis that the topology of the cerebral networks could unveil abnormal features in both prodromal and over dementia stage of AD. It is expected that abnormalities of the features would regard the presence of hubs, the measures of segregation, of integration and of resilience in groups of AD-MCI, and ADD patients. In addition, the inclusion of the direction of the edges between electrode pairs could provide a better understanding of the organizational properties of brain network in AD-MCI and in ADD patients compared to healthy subjects.

## Methods

### Study Population

The control subjects and the patients gave their written consent to the use of the unidentified results of their clinical, instrumental and laboratory investigations for research purposes. All study procedures were carried out in concordance with the Declaration of Helsinki and were approved by the Local Ethics Committee. Control group, ADD patients, and AD-MCI were recruited at the Clinical Neurology Unit, Department of Neuroscience (DINOGMI), University of Genoa, Italy. They underwent clinical and neuropsychological evaluations to assess language, executive functions, visuospatial abilities, verbal memory, attention and working memory, according to the neuropsychological test battery reported elsewhere (Picco et al. 2014).

The AD-MCI patients were retrospectively selected with the only criterion to be followed-up until the development of dementia of the AD type (ADD). To the purpose of this study, MCI patients who were stable at follow-up or developed dementia other than AD were not considered. Moreover, the selected MCI patients showed at least one positive neurodegeneration biomarkers according to the 2011 NIA-AA criteria (Albert et al. 2011). Biomarkers of amyloidosis were not available yet both at baseline evaluation and at follow-up diagnosis. Specifically, MCI patients had a characteristic pattern of altered metabolism seen at FDG-PET (64% of patients) or seen at perfusion single-photon emission computed tomography (SPECT; 26% of patients); hippocampal atrophy revealed by MRI (57% of patients) or computed tomography scans (24% of patients). Presence of an imaging biomarker of AD-neurodegeneration but lack of amyloidosis biomarkers qualifies these patients as affected by AD-MCI with intermediate likelihood according to Albert et al. (2011). However, ADD was confirmed in all patients at follow-up as per inclusion criteria. MCI patients showed impairment in a memory test (i.e., Rey auditory verbal learning test or Babcock story recall), either with (multi-domain amnestic MCI) or without (single-domain amnestic MCI) involvement of other cognitive domains, but did not meet criteria for dementia (Petersen and Negash 2008). A mini mental state examination (MMSE) score ≥ 24 and a 15-item geriatric depression scale score ≤ 10 were considered necessary to MCI diagnosis.

The presence of dementia in AD patients was established by clinical interviews with the patient and caregivers, by activities of daily living (ADL) and instrumental ADL questionnaires, and by the clinical dementia rating (CDR) scale and the MMSE. Only patients with MMSE score ≥ 20 (mild dementia) attributed to AD according to the international criteria were included in the study (McKhann et al. 2011). As in the case of AD-MCI patients, at least one neurodegeneration biomarker disclosed a typical AD pattern, among perfusion SPECT, FDG-PET, or MRI.

The healthy condition of the control subjects was carefully checked by means of general medical history and clinical examination. Only subjects with a normal MMSE score (i.e., > 26) and with a CDR of 0 were included. Brain MRI or CT were available in all control subjects and did not disclose major abnormalities, including medial temporal lobe atrophy. Given these prerequisites, the control subjects were chosen with the selection criteria of being in the same age range, having similar gender distribution and educational level as patients.

Exclusion criteria for patients and controls were: previous or present major psychiatric/neurological disease, severe and uncontrolled arterial hypertension, diabetes mellitus, renal, hepatic or respiratory failure, anaemia and malignancy.

The study population consisted of 83 patients (42 were classified as AD-MCI and 41 as ADD) and 42 control subjects.

### EEG Recordings

EEG was recorded with Ag/AgCl disk scalp electrodes from 19 scalp derivations placed according to the international 10–20 system and two additional electrodes placed on right and left earlobe. Linked earlobes were used as reference and sampling rate was 256 Hz.

Any drug, caffeine, nicotine and alcohol prohibition were withdrawn for at least 48 h prior to neuropsychological and neurophysiological assessment.

Recordings were obtained with subjects resting comfortably, with their eyes closed. Patients’ wakefulness was ascertained every 3 min inviting them to open their eyes. A simultaneous electrooculogram was recorded and muscular or tremor artefacts were controlled with supplementary derivations. Two pairs of bipolar recording channels for respiration and electrocardiogram were also applied. EEG was acquired as a continuous signal for 30 min and visually inspected for current clinical interpretation or detection of artefacts. Nineteen electrodes from Fp1, Fp2, Fz, F3, F4, F7, F8, Cz, C3, C4, Pz, P3, P4, T3, T4, T5, T6, O1, and O2 were considered for the analysis.

### GC Analysis

For each subject EEG recording was visually inspected to select artefact free 10 epochs of 4096 time points (16 s) long. The epochs could be also non-consecutive. Temporal filters were not applied to EEG recordings to minimize difficulties in model fitting (Barnett and Seth 2014). Time domain GC connectivity analysis was applied on each epoch to identify patterns of causal interaction between electrodes.

According to linear vector autoregressive (VAR) models, two wide-sense stationary time series X(t) and Y(t) can be explained by their own past by means of a linear model with coefficients a_j_ and b_j_ and prediction errors ε_1_(t) and η_1_(t), respectively:

1 $$X(t)=\sum\limits_{{j=1}}^{m} {{a_j}X(t - j)+{\varepsilon _1}(t)} ,$$

2 $$Y(t)=\sum\limits_{{j=1}}^{m} {{b_j}Y(t - j)+{\eta _1}(t)} .$$

Lagged vector autoregression models are used to determine the ability of one time-varying signal to predict the future behaviour of another, comparing the accuracy of the prediction obtained by considering only information of the signal own past with the prediction obtained by including the past of another signal of the system (Granger 1969). If the prediction error of the VAR model results to be higher than the prediction error obtained including another signal, then it is more accurate to describe the temporal dynamics of the time series X(t) and Y(t) (both of length T) including in the model information the past of the other time series, since the prediction errors ε_2_(t) and η_2_(t) are lower than the previous ε_1_(t) and η_1_(t).

3 $$X(t)=\sum\limits_{{j=1}}^{m} {{a_j}X(t - j)+\sum\limits_{{j=1}}^{m} {{b_j}Y(t - j)+{\varepsilon _2}(t)} },$$

4 $$Y(t)=\sum\limits_{{j=1}}^{m} {{c_j}Y(t - j)+\sum\limits_{{j=1}}^{m} {{d_j}X(t - j)+{\eta _2}(t)} } ,$$

where m is the maximum number of lagged observations included in the model (the model order, m ≪ T), whereas b_j_ and d_j_ are the gain factors, respectively, of the signal Y(t) (driver) influencing the signal X(t) (recipient), and of the signal X(t) (driver) influencing the signal Y(t) (recipient).

The linear influence from X(t) to Y(t) (F_X → Y_) and from Y(t) to X(t) (F_Y → X_) can be calculated as the log ratio between the variances of the residual errors.

5 $${F_{X \to Y}}=\log \left( {\frac{{\operatorname{var} ({\eta _1})}}{{\operatorname{var} ({\eta _2})}}} \right),$$

6 $${F_{Y \to X}}=\log \left( {\frac{{\operatorname{var} ({\varepsilon _1})}}{{\operatorname{var} ({\varepsilon _2})}}} \right).$$

GC magnitude is given by the log ratio of the variance of the prediction-error terms for the reduced (omitting the signal of the potential cause) and full regressions (including the signal of the potential cause).

GC analysis is generalized to the multivariate (conditional) case in which the GC of Y(t) on X(t) is tested in the context of multiple additional variables (Geweke 1982) when all other variables are also included in the regression model.

In this study GC analysis was performed using the in house software BSMART, a MATLAB/C toolbox implemented to analyse brain circuits (Cui et al. 2008). A conditional multivariate vector autoregression (MVAR) model was applied to the 19 time series from the 19 electrodes to estimate GC connectivity (Seth 2010). The method of ordinary-least-squares was used to compute the regression coefficients. The F-statistic, Bonferroni-corrected (nominal p value of 0.05, then divided for multiple comparisons by n, where n = 19), was applied to the coefficients of the MVAR model. When they did not reach the significant threshold the corresponding GC magnitude was set to zero. The Akaike information criterion (1974) was used to estimate the order of the model (Bressler and Seth 2011) for each subject and epoch, separately. When the Akaike information criterion did not find a global minimum, the epoch was discarded. Covariance stationarity of each epoch was checked by using the Durbin–Watson test, based on MATLAB code provided by Seth (2010) and the Dickey–Fuller test (p < 0.01) to identify unit roots. The consistency of the MVAR model, which ensures that the MVAR model properly represents the data, was verified by the tests proposed by Ding et al. (2000) and by the Durbin–Watson statistics, which assess whether the residuals are uncorrelated. Epochs with model consistency lower than 80% were discarded.

GC analysis was computed for each subject and epoch, separately, by a MATLAB toolbox for multi-trial data (Seth 2010), obtaining a GC matrix of 19 rows and 19 columns of GC magnitude, representing the causal strength of the connection between each couple of vertices. Finally, for each subject the 10 GC matrices were averaged, and the mean values of GC magnitude (causal strength) for each connection between 19 vertices were used as weighted directed connectivity matrix for the graph theory analysis. For each subject the weighted directed connectivity matrix was not thresholded.

### Graph Theory Parameters

The Brain Connectivity Toolbox codebase (http://www.brain-connectivity-toolbox.net) was used to estimate graph theory parameters (Rubinov and Sporns 2010) on GC connectivity matrix.

From a wide set of parameters, we selected the most useful to characterize the brain network of control group, AD-MCI and ADD patients (Pavlopoulos et al. 2011).

Networks can be characterized at different levels ranging from the global scale to the local scale. Starting from the local scale, the components of a network are its vertices and edges. The degree of a vertex i (K_i_) is the sum of its incoming (afferent) and outgoing (efferent) edges (a_ij_):

7 $${K_i}=\sum\limits_{{j=1}}^{n} {{a_{ij}}} ,$$

where a_ij_ = 1 when the link between i and j exists, a_ij_ = 0 otherwise.

To avoid ambiguity with directed links each undirected link was counted twice.

The number of afferent and efferent connections is also called the in-degree and out-degree, respectively. By means of GC approach is possible to distinguish incoming and outgoing edges, so that in-degree and out-degree were also calculated. Vertices with a high number of edges, i.e., a large degree, are called network hubs. Vertices with predominantly incoming edges can be seen as sinks (integrators, convergence) whereas vertices with mainly outgoing edges can be seen as sources (distributors, divergence) or broadcasters of information. These distinctions can be useful when vertices are otherwise similar, e.g., distinguishing different types of network hubs (Sporns et al. 2007).

The clustering coefficient and the local efficiency were used as measures of segregation.

The clustering coefficient of the vertex i is defined as

8 $${C_i}=\frac{{2{t_i}}}{{{K_i}({K_i} - 1)}},$$

where

9 $${t_i}=\frac{1}{2}\sum\limits_{{j,k}} {{a_{ij}}{a_{ik}}{a_{jk}}}$$

is the number of triangles around a vertex i.

Simple measures of segregation are based on the number of triangles in the network, with a high number of triangles implying segregation. All vertices that are connected to a vertex by a direct edge are defined as neighbours of that vertex. Locally, the fraction of triangles around an individual vertex is known as the clustering coefficient and is equivalent to the fraction of the vertex neighbours that are also neighbours with each other (Watts and Strogatz 1998). The clustering coefficient is a measure of the tendency of network elements to form local clusters (de Haan et al. 2009) and can help defining if the vertices tend to form cliques, or small groups of closely interconnected vertices. High clustering is associated with robustness of a network, i.e., resilience against damage.

The local efficiency plays a role similar to the clustering coefficient. It is defined as the average efficiency of the local subgraphs. The local efficiency of the vertex i (E_loc,i_) is defined as

10 $${E_{loc,i}}=\frac{{\sum\nolimits_{{j,h}} {{a_{ij}}{a_{ih}}{{({d_{jh}})}^{ - 1}}} }}{{{K_i}({K_i} - 1)}},$$

where d_jh_ is the length of the shortest path between j and h, that contains only neighbours of i.

This quantity reveals how much the system is tolerant to faults (Latora and Marchiori 2010), thus it shows how efficient the communication is between the first neighbours of the vertex when this vertex is removed.

Average shortest path length (also called the characteristic path length) and global efficiency were evaluated as measures of integration. The characteristic path length (L) is defined as

11 $$L=\frac{1}{n}\sum\limits_{i} {\frac{{\sum\nolimits_{{j \ne i}} {{d_{ij}}} }}{{n - 1}}},$$

where n is the number of vertices.

A measure of travelling through a network is the number of edges one has to cross, on average, to go from one vertex to another. The average shortest path of a network is the average number of edges that has to be crossed on the shortest path from any one vertex to another. The average shortest path length to any other vertex is calculated and the median value over all vertices is returned as the characteristic path length. The average shortest path only takes the existing shortest paths between pairs of vertices so that it is primarily influenced by existing paths.

Global efficiency (E) is a sum of the inverse of the characteristic path length. Thus, it is defined as

12 $$E=\frac{1}{n}\sum\limits_{i} {\frac{{\sum\nolimits_{{j \ne i}} {d_{{ij}}^{{ - 1}}} }}{{n - 1}}}.$$

It may be meaningfully computed on disconnected networks, as paths between disconnected vertices are defined to have infinite length, and correspondingly zero efficiency, so it is primarily influenced by short paths. Some authors have argued that this may make the global efficiency a superior measure of integration (Achard and Bullmore 2007).

The assortativity coefficient was performed as a measure of resilience. The assortativity coefficient is a correlation coefficient between the degrees of all vertices on two opposite ends of a edge. It is defined as

13 $$r=\frac{{{l^{ - 1}}\sum\nolimits_{{i,j}} {{K_i}{K_j} - {{\left[ {{l^{ - 1}}\sum\nolimits_{{i,j}} {\frac{1}{2}({K_i}+{K_j})} } \right]}^2}} }}{{{l^{ - 1}}\sum\nolimits_{{i,j}} {\frac{1}{2}({K_i}^{2}+{K_j}^{2}) - {{\left[ {{l^{ - 1}}\sum\nolimits_{{i,j}} {\frac{1}{2}({K_i}+{K_j})} } \right]}^2}} }},$$

where l is the number of links.

Networks with a positive assortativity coefficient are therefore likely to have a comparatively resilient core of mutually interconnected high-degree hubs. On the other hand, networks with a negative assortativity coefficient are likely to have widely distributed and consequently vulnerable high-degree hubs.

### Statistical Analysis

Demographic and clinical differences among groups were assessed with analysis of variance (ANOVA) for continuous data and χ^2^ test for categorical data.

Since not all graph theory variables showed a Gaussian distribution (Kolmogorov–Smirnov test), network parameters comparisons among the three groups were performed using nonparametric statistics (Kruskal–Wallis test followed by Dunn’s post hoc tests when appropriate, adjusted p value for multiple comparison tests was performed using the Bonferroni error correction). Kruskal–Wallis test was performed on each of the 19 vertices to compare degree, in-degree, out-degree, clustering coefficient and local efficiency among groups.

Wilcoxon signed ranks test was used to compare in-degree and out-degree values within each group separately. To estimate the association between AD disease severity and network organization, we calculated the Spearman correlation between MMSE scores of patients and network measures. The bias-corrected and accelerated bootstrap method (Ruscio 2008) with 1000 iterations was used to construct 95% confidence interval (CI) for significant correlations. In addition, Spearman correlation was also performed between age and graph theory parameters in control and patient groups.

## Results

### Study Population

Control group, AD-MCI, and ADD patient groups did not differ for age, gender and educational level (p > 0.05). Table 1 reports all mean values, standard deviations, and statistical results.

**Table 1:** Demographic and clinical characteristics of the three groups

	Control n = 42	AD-MCI n = 42	ADD n = 41	Statistical results
Age^a^	73.7 ± 7.4	74.8 ± 7.8	77.3 ± 6.1	F(2,122) = 2.6, p = 0.08
Gender (% male)^b^	50	38	39	χ^2^ = 1.5, p = 0.47
Educational level^a^	9.9 ± 3.3	9.2 ± 4.5	8.2 ± 4.4	F(2,122) = 2.5, p = 0.08
MMSE^a^	28.9 ± 0.9	25.0 ± 1.0	21.5 ± 1.1	F(2,122) = 631.6, p < 10^− 4^

Data are presented as mean ± standard deviation

*ADD* Alzheimer’s disease with dementia, *AD-MCI* mild cognitive impairment due to Alzheimer’s disease, *MMSE* mini-mental state examination

^a^ANOVA analysis. Duncan post-hoc comparisons for MMSE. MMSE higher in control than in AD-MCI (p = 10^− 4^) and ADD (p = 10^− 4^). MMSE was higher in AD-MCI than ADD (p = 10^− 4^)

^b^Kruskal–Wallis test

### Graph Theory

Each GC connectivity matrix on a single subject level was used to estimate graph theory parameters: degree, in-degree, out-degree, measures of segregation, integration and resilience computed from EEG data at electrode pairs (Pavlopoulos et al. 2011).

**Fig. 1:**
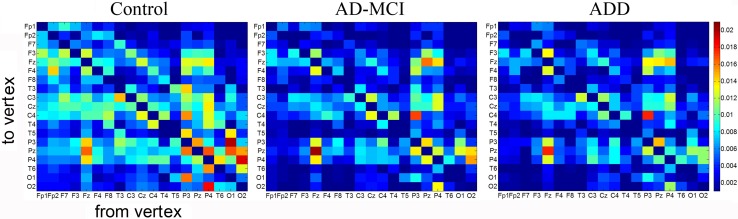
Mean GC magnitudes across subjects for all links in control, AD-MCI and ADD groups. In the matrix representation “from vertex to vertex” indicates the direction of the information transfer between electrode pairs. Electrodes are shown from left to right side, anterior-posteriorly. The color bar indicates the magnitude of GC connections. Of note the number of drivers which have high values of effective connectivity (in red) is higher in control group than in AD-MCI and ADD. The effective connectivity was higher in control than AD-MCI and ADD for several edges: from P3 to O1, Pz, from P4 to O2, T6, Pz, from O1 to Pz, T3, T5, from O2 to T6, P4, Pz, etc.

Figure 1 shows mean GC connectivity matrices from EEG across subjects for control, AD-MCI, and ADD groups. The figure highlights that the number of drivers with high values of effective connectivity (in red) is larger in the control group than the AD-MCI and ADD groups.

### Degree, In-degree, Out-degree

Degree was different among groups for frontal (F4, F7, F8), temporal (T3, T4–6), central (Cz, C4), parietal (P3, Pz, P4) and occipital (O1, O2) electrodes. Median values, ranges and statistical results on the comparison among groups were reported in Table 2. We considered as hubs electrodes, only high-degree vertices (i.e., vertices with a degree at least one standard deviation above the network mean) (Sporns et al. 2007). Thus for all groups, degree values showed that Fz, C3, C4, Cz, P3, P4 and Pz electrodes could be defined as hubs. F3 electrode could be considered as hub for patients’ groups only.

**Table 2:** Degree and statistical comparisons among groups

Vertex	Degree			Main effect	p value from post-hoc	
	Control	AD-MCI	ADD	H(2,125)	Controls versus AD-MCI	Controls versus ADD
Fp1	8 (0–26)	5 (0–26)	3 (0–26)	H = 4.93, p = 0.09	n.a.	n.a.
Fp2	7 (0–25)	4 (0–25)	3 (0–26)	H = 6.92, p = 0.03	n.s.	n.s.
F7	8 (1–34)	5 (0–25)	6 (0–27)	H = 7.42, p = 0.03	0.04	n.s.
F3	15 (1–35)	9 (0–29)	10 (1–27)	H = 4.47, p = 0.11	n.a.	n.a.
**Fz**	18 (7–33)	15 (3–30)	15 (2–29)	H = 7.40, p = 0.03	n.s.	n.s.
F4	14 (0–33)	9 (0–28)	9 (0–29)	H = 8.32, p = 0.02	n.s.	0.03
F8	10 (0–31)	4 (0–35)	6 (0–23)	H = 12.37, p = 0.002	0.003	0.02
T3	9 (0–30)	4 (0–25)	7 (0–26)	H = 11.46, p = 0.003	0.003	n.s.
**C3**	17 (2–31)	12 (2–29)	14 (2–28)	H = 4.45, p = 0.11	n.a.	n.a.
**Cz**	18 (8–31)	13 (5–31)	15 (4–33)	H = 10.66, p = 0.005	0.01	0.02
**C4**	18 (3–30)	13 (2–27)	14 (1–28)	H = 8.84, p = 0.01	0.02	n.s.
T4	10 (0–30)	6 (0–24)	5 (0–29)	H = 9.44, p = 0.009	0.01	n.s.
T5	9 (0–31)	4 (0–21)	4 (0–22)	H = 8.86, p = 0.01	0.03	0.04
**P3**	21 (4–32)	16 (0–31)	14 (3–27)	H = 13.04, p = 0.001	0.004	0.007
**Pz**	20 (8–34)	18 (6–30)	16 (6–33)	H = 8.90, p = 0.01	n.s.	0.01
**P4**	22 (11–33)	16 (4–32)	17 (1–28)	H = 13.39, p = 0.001	0.004	0.006
T6	9 (1–29)	4 (0–26)	4 (0–19)	H = 13.94, p = 0.001	0.002	0.01
O1	9 (1–29)	6 (2–22)	6 (0–22)	H = 9.14, p = 0.01	0.03	0.03
O2	9 (2–31)	7 (2–31)	7 (0–27)	H = 7.81, p = 0.02	0.04	n.s.

For each vertex, values are medians, with range printed between parentheses. Hub vertices for all groups are in bold. F3 is a hub for patients’ groups. For each vertex, main effect results and adjusted p values for multiple comparisons are shown for post-hoc analysis

*ADD* Alzheimer’s disease with dementia, *AD-MCI* mild cognitive impairment due to Alzheimer’s disease, *n.a*. not applicable following not significant main effect, *n.s*. not significant

In-degree was different among groups for frontal (F4, F8), central (C3, C4, Cz), parietal (P3, P4, Pz), temporal (T3–6) electrodes and O1. Out-degree was different among groups for frontal (F7, F8), parietal (P3, P4), temporal (T3, T6) electrodes and O1. All statistical results on in-degree and out-degree were reported in Table 3.

**Table 3:** In-degree, out-degree statistical comparisons among groups

Vertex	In-degree			Out-degree		
	Main effect	p value from post-hoc		Main effect	p value from post-hoc	
	H(2,125)	Controls versus AD-MCI	Controls versus ADD	H(2,125)	Controls versus AD-MCI	Controls versus ADD
Fp1	H = 2.87, p = 0.2	n.a.	n.a.	H = 6.23, p = 0.04	n.s.	n.s.
Fp2	H = 5.70, p = 0.06	n.a.	n.a.	H = 6.52, p = 0.04	n.s.	n.s.
F7	H = 4.70, p = 0.1	n.a.	n.a.	H = 10.02, p = 0.007	0.02	0.02
F3	H = 3.06, p = 0.2	n.a.	n.a.	H = 5.38, p = 0.07	n.a.	n.a.
Fz	H = 6.36, p = 0.04	n.s.	n.s.	H = 5.67, p = 0.06	n.a.	n.a.
F4	H = 6.96, p = 0.03	n.s.	0.03	H = 6.61, p = 0.04	n.s.	n.s.
F8	H = 9.87, p = 0.007	0.01	0.03	H = 9.99, p = 0.007	0.007	n.s.
T3	H = 6.85, p = 0.03	0.03	n.s.	H = 12.53, p = 0.002	0.001	n.s.
C3	H = 7.38, p = 0.03	0.047	n.s.	H = 1.42, p = 0.5	n.a.	n.a.
Cz	H = 9.19, p = 0.01	0.01	n.s.	H = 10.66, p = 0.005	0.01	0.02
C4	H = 13.11, p = 0.001	0.002	0.022	H = 2.26, p = 0.3	n.a.	n.a.
T4	H = 10.74, p = 0.005	0.003	n.s.	H = 7.25, p = 0.03	n.s.	n.s.
T5	H = 11.88, p = 0.003	0.005	0.02	H = 6.22, p = 0.045	n.s.	n.s.
P3	H = 14.41, p = 0.001	0.003	0.003	H = 7.30, p = 0.03	0.04	n.s.
Pz	H = 11.65, p = 0.003	0.04	0.003	H = 3.95, p = 0. 1	n.a.	n.a.
P4	H = 12.40, p = 0.002	0.007	0.007	H = 7.63, p = 0.02	0.04	n.s.
T6	H = 11.28, p = 0.004	0.006	0.02	H = 11.78, p = 0.003	0.006	0.02
O1	H = 8.02, p = 0.02	0.04	0.046	H = 8.85, p = 0.01	0.02	0.05
O2	H = 7.44, p = 0.02	n.s.	n.s.	H = 4.09, p = 0.1	n.a.	n.a.

For each vertex, main effect results and adjusted p values for multiple comparisons are shown for post-hoc analysis

*ADD* Alzheimer’s disease with dementia, *AD-MCI* mild cognitive impairment due to Alzheimer’s disease, *n.a*. not applicable following not significant main effect, *n.s*. not significant

Degree, in-degree and out-degree values were higher in control than in patient’s groups. No difference was found between the AD-MCI and the ADD group. No correlation was found between degree and either MMSE or age.

**Fig. 2:**
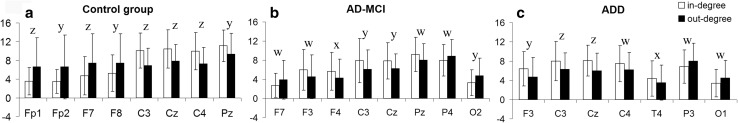
In-degree and out-degree values for vertices which showed significant differences inside control (**a**), AD-MCI (**b**) and ADD (**c**) groups. P values are shown by the letters “w” indicating p < 0.05, “x” p < 0.01, “y” p < 0.005 and “z” p < 0.001

Wilcoxon signed ranks test between in-degree and out-degree values showed significant differences in frontal (Fp1, Fp2, F7, F8), central (C3, C4, Cz), Pz electrodes for control group, in frontal (F3, F4, F7), central (C3, Cz), parietal (P4, Pz) and O2 electrodes for AD-MCI group, in central (C3, C4, Cz), F3, P3, T4, O1 electrodes for ADD group (Fig. 2). Specifically, for control group (Fig. 2a), frontal electrodes had predominantly outgoing edges (out-degree values higher than in-degree values), whereas central electrodes and Pz, had predominantly incoming edges (in-degree values higher than out-degree values). For AD-MCI (Fig. 2b), F7, P4 and O2 had predominantly outgoing edges, whereas F3, F4, C3, Cz and Pz had predominantly incoming edges. For ADD (Fig. 2c), P3 and O1 had predominantly outgoing edges, whereas F3, C3, C4, Cz and T4 had predominantly incoming edges. All significant p values are shown in Fig. 2.

### Measure of Segregation

**Fig. 3:**
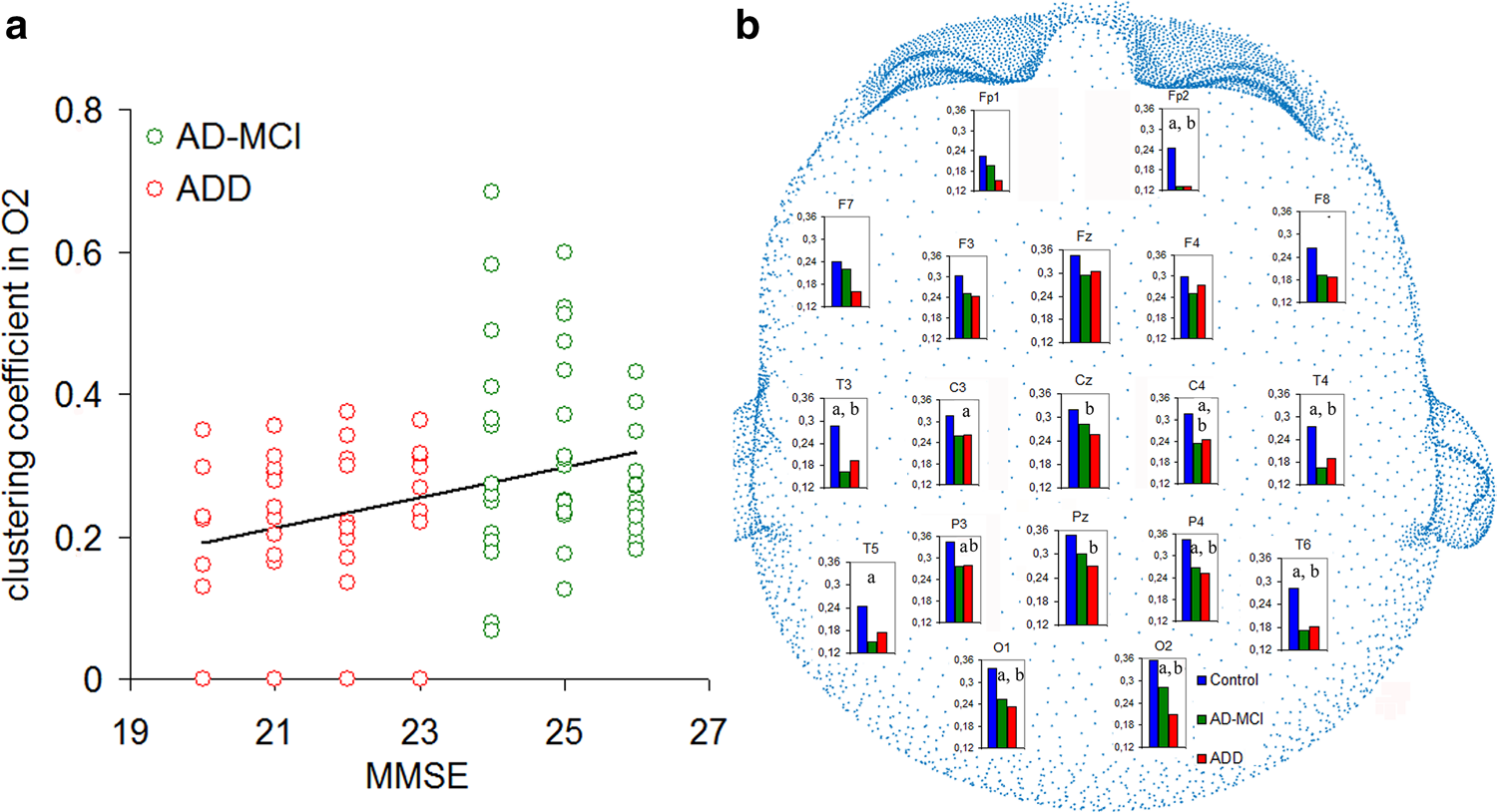
Measures of segregation. **a** Correlation between clustering coefficient and MMSE in patients’ groups (Spearman ρ = 0.286, p = 0.009). **b** Mean local efficiency for control (blue bars), AD-MCI (green) and ADD (red) group. Significant differences between control and AD-MCI, control and ADD, AD-MCI and ADD are shown with a, b and c, respectively

The statistical comparisons among groups on clustering coefficient of the 19 vertices showed significant main effect for O2 [H(2,125) = 6.76, p = 0.03]. Clustering coefficient was higher in AD-MCI than ADD for O2 (p = 0.028). Positive correlation was found between the clustering coefficient in O2 and MMSE in the patients’ groups (Spearman ρ = 0.286, p = 0.009, CI = [0.09, 0.46], Fig. 3a). As control analysis we also performed the clustering coefficient using binary directed GC connectivity matrix (GC magnitude were binarized, all connections have equal strength 0 or 1). Then, we re-performed the correlation between clustering coefficient in O2 and MMSE for the patients groups. Spearman correlation revealed a significant positive correlation between clustering coefficient in O2 and MMSE (ρ = 0.275, p = 0.01, CI = [0.06, 0.47]).

Local efficiency was different among groups in Fp2, central (C3, C4, Cz), parietal (P3, P4, Pz), temporal (T3–6) and occipital (O1, O2) electrodes. Local efficiency was higher in control than in AD-MCI and ADD group. All significant statistical results on local efficiency were reported in Table 4.

**Table 4:** Local efficiency statistical comparisons among groups

Vertex	Local efficiency		
	Main effect	p value from post-hoc	
	H(2,125)	Controls versus AD-MCI	Controls versus ADD
Fp2	H = 11.72, p = 0.003	0.008	0.01
T3	H = 15.62, p = 0.001	0.001	0.01
C3	H = 8.24, p = 0.02	0.03	n.s.
Cz	H = 8.09, p = 0.02	n.s.	0.01
C4	H = 9.96, p = 0.007	0.01	0.04
T4	H = 13.35, p = 0.001	0.001	0.02
T5	H = 9.36, p = 0.009	0.008	n.s.
P3	H = 9.77, p = 0.008	0.03	0.02
P4	H = 13.34, p = 0.001	0.02	0.002
T6	H = 16.63, p = 0.001	0.001	0.003
O1	H = 12.83, p = 0.002	0.02	0.002
O2	H = 22.64, p = 0.001	0.02	0.001

For each vertex, main effect results and adjusted p values for multiple comparisons are shown for post-hoc analysis

*ADD* Alzheimer’s disease with dementia, *AD-MCI* mild cognitive impairment due to Alzheimer’s disease, *n.s*. not significant

Mean local efficiency values across subjects and the significant differences across groups were shown in Fig. 3b for each vertex. No correlation was found between the local efficiency and both MMSE and age.

### Measure of Integration

Characteristic path length was similar among groups. It tended to be higher in the patients’ groups than control group, but the increase was not significant. No correlation was found between characteristic path length and either MMSE or age.

The global efficiency was different among groups [H(2,125) = 13.7, p = 0.001]. It was higher in the control than the AD-MCI (p = 0.01) and the ADD group (p = 0.002). No difference was found in the comparison between the AD-MCI and ADD groups. No correlation was found between the global efficiency and both MMSE and age.

### Measure of Resilience

Assortativity coefficients for strength were not different among groups. Assortativity coefficient for out–out degree was different among groups [H(2,125) = 6.27, p = 0.04]. It was lower (more negative) in ADD than control group (p = 0.045).

Assortative coefficients for degree were negative for all the groups. A negative assortativity coefficient indicates that inside the network the vertices characterized by a high number of edges tend to be connected to vertices with low number of edges (dissortative network). No correlation was found between assortativity and either MMSE or age.

## Discussion

Previous resting state EEG studies on functional organization of the cerebral network in AD as revealed by graph theory reported conflicting results. These discrepancies among studies could be related to methodological differences. The estimation of the direction of the edges allows to disentangle some issues, distinguishing the contribution of the incoming and outgoing information transfer from or to a vertex of the network. For this purpose, in the present study, the MVAR model applied to GC analysis was used as an effective connectivity measure of the EEG signal applied to graph theory. The MVAR model avoids the pitfalls connected with the application of bivariate EEG measures between electrode pairs (Blinowska 2011). GC analysis was used instead of effective connectivity based on phase differences of the EEG rhythms. Indeed it was shown that such measures can give erroneous estimates of direction of information flow in the resting state EEG rhythms (Hillebrand et al. 2016).

GC analysis was applied in the time domain because it has the advantage not to have a-priori hypothesis on the frequency bands of the information transfer. Effective connectivity measures at a given frequency band of the resting state EEG rhythms can potentially represent frequency-specific brain oscillatory processes only. They did not take into account the inter-subjects’ variability of that frequency-specific brain oscillatory processes, including that induced by AD. In addition effective connectivity in frequency domain unveils only information transfers in the same frequency bands and the possible information transfers which changed frequency bands are not evaluated.

In the present study, graph theory parameters were weighted by the strength of the edges revealed by GC results. GC results showed the reduction of the strength of the information transfer from resting state EEG signal in the AD-MCI and ADD than the control group for bilateral edges among parieto-temporal and occipital areas (Fig. 1). Compared with control subjects, previous studies showed that ADD patients were typically characterized by a decrease in the resting state alpha coherence between electrode pairs (Knott et al. 2000; Pogarell et al. 2005), with main effects in temporo–parieto-occipital regions (Locatelli et al. 1998; Adler et al. 2003). Directed transfer function study displayed a reduction in alpha and beta strength from parietal to frontal electrodes in ADD and MCI patients compared with control subjects (Koenig et al. 2005; Babiloni et al. 2008, 2009; Dauwels et al. 2010b).

### Degree, In-degree, Out-degree

Degree values showed an overall decrease of edges in patients’ groups than control group. This result is in agreement with previous EEG and magnetoencephalographic studies reporting a decrease of resting state functional connectivity in AD patients (Knott et al. 2000; Koenig et al. 2005; Franciotti et al. 2006; Stam et al. 2009). AD-MCI and ADD showed a lower number of edges among the vertices than the control group for temporal electrodes, supporting the hypothesis that the number of edges among brain areas is reduced since the MCI stage of AD. GC magnitude was also reduced in temporal electrodes of patients (Fig. 1). This functional alteration could be related to medial temporal lobe atrophy which is a characteristic neuropathological change in the early stages of AD (Rusinek et al. 2004; Mistur et al. 2009). Previous EEG studies showed that power alterations in theta and beta bands were related to temporal atrophy (Lee et al. 2015) and slowing of the background activity was found to be more prominent in temporal derivations (Valladares-Neto et al. 1995). A FDG-PET study reported that amnestic MCI patients, who eventually developed AD, showed significant hypo-metabolism in the left middle and superior temporal gyri (Morbelli et al. 2010). Another study reported oxidative damage to the superior temporal gyrus (STG) during the prodromal stage of AD, suggesting that oxidative damage to the temporal lobe is an early event in the onset of AD (Keller et al. 2005). Abnormal functional connectivity of the STG was also found in MCI patients studied by functional MRI (Risacher et al. 2009; Davatzikos et al. 2011) and by means of FDG-PET (Morbelli et al. 2012). By means of phase synchronization estimation, a decrease in alpha 2 lagged phase synchronization between temporal and parietal electrodes was shown in ADD patients compared with control subjects (Canuet et al. 2012) and an increase of delta band phase synchronization was found in ADD revealing that the temporal lobe connections were particularly compromised (Canuet et al. 2012).

Although the number of edges was lower in patients’ groups than control group, the vertices with highest degree (so called hubs) were Fz, C3, C4, Cz, P3, P4 and Pz for all groups, suggesting that the hub function was not abnormal in the prodromal and mild dementia stages of AD. Degree showed that the number of edges was preserved for frontal electrodes and F3 represented a hub in patient’s groups. These results extend the findings of a previous study on correlation coefficients between pairs of gray matter regions obtained by MRI, where the MCI and AD groups retained their hub regions in the frontal lobe, as compared to healthy controls (Yao et al. 2010).

By means of graph theoretical analysis applied to GC matrices, in-degree and out-degree variables were compared among groups. The reduction of the in-degree and out-degree variables was found in both AD-MCI and ADD as compared to control group. In addition, the topology of the network was different among groups. Specifically, in the control group frontal electrodes could be seen as broadcaster of information flows whereas central electrodes as integrator showing higher values of out-degree than in-degree and in-degree than out-degree, respectively (Fig. 2a). For the patients’ groups the pattern was reversed: the frontal electrodes could be seen as integrators, whereas parieto-occipital electrodes as broadcasters (Fig. 2b, c). For the control group the higher number of incoming edges found in hubs like C3, C4, Cz and Pz extend a previous EEG study (Moon et al. 2015) which reported that hubs have a more receiving role in the network compared to non-hubs. Instead in AD-MCI and ADD this receiving role of the hubs was altered for P4 and P3 which had more outgoing than incoming connections. These results suggest a different functional organization of the parietal derivations in AD-MCI and ADD group compared with control group, confirming the hypothesis of an affected pattern of information flow in the large-scale brain networks.

### Measure of Segregation

No difference was found between control and patients’ groups for the weighted directed clustering coefficient. Previous studies reported increased (He et al. 2008; Yao et al. 2010; Zhao et al. 2012), decreased (Stam et al. 2009; Tijms et al. 2013) or unmodified (Stam et al. 2007; Lo et al. 2010; Sanz-Arigita et al. 2010) clustering coefficient in AD compared to control group. The clustering coefficient was instead reduced in ADD compared with AD-MCI in O2. This reduction was found to be correlated to the cognitive impairment assessed by MMSE and to be independent from the GC magnitude of the connectivity matrix because significant correlation between clustering coefficient and MMSE was also found using binary coefficients of the GC correlation matrix. The amplitude of occipital sources of resting state alpha rhythms was found to be correlated to both MMSE scores and occipital gray matter density measured by MRI in MCI and AD patients (Babiloni et al. 2015). Thus our results could be related to the relationship between the occipital alpha rhythms, AD neurodegeneration in the occipital lobe and cognitive status.

The occipital electrodes showed higher values of local efficiency in control group than patients’ groups (Fig. 3b). GC magnitudes in O1 and O2 (Fig. 1) suggest the involvement of these vertices in the formation of cliques in the control and AD-MCI groups. Our results on measures of segregation suggest that brain network functional alterations mainly involve the parieto-temporal derivations in the MCI stage of AD, whereas brain dynamic changes in the occipital electrodes are evident in AD with overt dementia.

### Measure of Integration

No differences among groups were found for the characteristic path length, whereas the global efficiency was higher in control than patients’ groups. Previous studies evidenced increased (Stam et al. 2007, 2009; He et al. 2008; Lo et al. 2010; Yao et al. 2010; Zhao et al. 2012) or decreased (Sanz-Arigita et al. 2010; Tijms et al. 2013) characteristic path length in AD. Our results suggest a reduction of the global efficiency of the network in AD-MCI and in AD with mild dementia and the possible preservation of the average path length of the information flow.

### Measure of Resilience

The negative values of the assortativity coefficient for the three groups suggest that the networks are dissortative (the vertices predominantly connect with other vertices of different degree) in the three groups. Biological networks tend to be dissortative (Newman 2002). An assortative network is generally associated with a more efficient information processing and a lower vulnerability to random network damage. Targeted attacks in assortative networks are highly effective if compared to random breakdowns, due to the critical high degree of just a few vertices (hubs) whose removal can disrupt the whole network (Boccaletti et al. 2006). In ADD, the reduction of the number of edges and of the out–out degree assortativity coefficient suggests an increased vulnerability of the network to both targeted and random attacks.

### Limitations

A major point of criticism on EEG and magnetoencephalography (MEG) is that the estimates of statistical interdependencies may be biased by the effects of volume conduction and, in the case of EEG, by the influence of the reference electrode (Nunez et al. 1997; Guevara et al. 2005).

While it is largely acknowledged that volume conduction and reference electrode deteriorate spatial resolution of scalp EEG, other distortions are less widely recognized in the community (Burle et al. 2015). Specifically, controversial findings were reported on the effect of volume conduction in the estimation of information flows derived from MVAR models. No influence of volume conduction was recognized when the estimators of connectivity are based on the phase difference between channels (Stam et al. 2009; Kaminski and Blinowska 2014). On the other hand spurious channel to channel connections or driver vertices influenced by higher signal–noise ratio were reported to be due to volume conduction (Haufe et al. 2011; Brunner et al. 2016).

A simulation study reported that the error rate on the EEG/MEG connectivity estimates due to the effects of volume conduction is less than 5.2% (Khadem and Hossein-Zadeh 2014). Thus, in our study the significant differences among groups on graph theory parameters could not be ascribed to volume conduction effects. However, further investigation should be performed using spatial filters such as Laplacian filters or image inverse methods to EEG data in order to reduce correlations among scalp-recorded channels (Baillet et al. 2001; Fisch 2012). In this study the use of low density EEG recordings did not allow a correct procedure of source estimation. Indeed the analysis on the source space needs an adequate coverage of both the superior and inferior surfaces and an adequate number of sensors because as sampling density increases, localization error decreases, regardless of the inverse method and head model (Song et al. 2015). In addition we did not apply the Laplace transform because this method mixing the information from different channels could destroy the original correlation structure between signals, and the causal information between channels is lost (Kaminski and Blinowska 2014).

The choice of the EEG reference is an important issue. Reference-free approaches or source localization should be applied to overcome this issue. For example, reference electrode standardization technique (Dong et al. 2017) seems to be a promising method (Chella et al. 2016; Huang et al. 2017), based on the assumption that an approximate neutral reference can be achieved at an infinity point which is far from brain sources. In this way the activated neuronal sources in the brain are always the same regardless of the reference schemes. Systematic studies should be performed with different methods to evaluate the influence of the EEG reference on graph theory estimations.

In addition GC results could be affected by the measurement noise, leading to spurious connection. Specifically, uncorrelated noise affects only weakly the detection of GC directionality, whereas linearly mixed noise causes a large fraction of false positives (Vinck et al. 2015). In general, the multivariate causality measures are very sensitive to data preprocessing (Florin et al. 2010). Extensive simulations were performed on the effect of applying different filtering techniques (high-pass, low-pass, notch filter) and four different filter types (Butterworth, Chebyshev I and II, elliptic filter), on the performance of multivariate causality measures. Results suggested that preprocessing without a strong prior about the artifact to be removed disturbs the information content and time ordering of the data and leads to spurious and missed causalities (Florin et al. 2010). However, future studies should be performed to improve GC performance in the presence of measurement noise.

Another limitation of the present study could be ascribed to the lack of a reference network for comparison. Our findings highlighted different network topologies among groups, but we can not conclude if the reduction of the edges or the decrease of the clustering coefficient linked to the MMSE actually reflect a loss of connections or a more random topology.

## Conclusions

GC results (direction and strength of the edges) in the whole frequency range applied to graph theory and a certified stage of prodromal AD (AD-MCI) are the main contributions of the present study. In both MCI and ADD conditions, the observed functional disconnections (Delbeuck et al. 2003) involve vertices with low and high degree.

A greater understanding of these early brain changes would inform the pathophysiology as well as be relevant to treatment trials targeting the early stages of AD (Brier et al. 2014).

Our results add new pieces of evidence in the comprehension of the progression of AD from the prodromal stage to dementia, suggesting that the functional network alteration, evident in AD patients also in their prodromal stage, begins with the reduction of the number of edges and the loss of local and global efficiency. These results confirm that by the time clinical symptoms are detected, at least some AD-related neurological damage has developed (Mosconi 2013).

## References

[CR1] Achard S, Bullmore E (2007). Efficiency and cost of economical brain functional networks. PLoS Comput Biol.

[CR2] Adler G, Brassen S, Jajcevic A (2003). EEG coherence in Alzheimer’s dementia. J Neural Transm (Vienna).

[CR3] Akaike H (1974). A new look at the statistical model identification. IEEE Trans Autom Control.

[CR4] Albert MS, DeKosky ST, Dickson D, Dubois B, Feldman HH, Fox NC, Gamst A, Holtzman DM, Jagust WJ, Petersen RC, Snyder PJ, Carrillo MC, Thies B, Phelps CH (2011). The diagnosis of mild cognitive impairment due to Alzheimer’s disease: recommendations from the National Institute on Aging-Alzheimer’s Association workgroups on diagnostic guidelines for Alzheimer’s disease. Alzheimers Dement.

[CR5] Babiloni C, Binetti G, Cassetta E, Dal Forno G, Del Percio C, Ferreri F, Ferri R, Frisoni G, Hirata K, Lanuzza B, Miniussi C, Moretti DV, Nobili F, Rodriguez G, Romani GL, Salinari S, Rossini PM (2006). Sources of cortical rhythms change as a function of cognitive impairment in pathological aging: a multi-centric study. Clin Neurophysiol.

[CR6] Babiloni C, Frisoni GB, Pievani M, Vecchio F, Infarinato F, Geroldi C, Salinari S, Ferri R, Fracassi C, Eusebi F, Rossini PM (2008). White matter vascular lesions are related to parietal-to-frontal coupling of EEG rhythms in mild cognitive impairment. Hum Brain Mapp.

[CR7] Babiloni C, Frisoni GB, Pievani M, Vecchio F, Lizio R, Buttiglione M, Geroldi C, Fracassi C, Eusebi F, Ferri R, Rossini PM (2009). Hippocampal volume and cortical sources of EEG alpha rhythms in mild cognitive impairment and Alzheimer disease. NeuroImage.

[CR8] Babiloni C, Del Percio C, Boccardi M, Lizio R, Lopez S, Carducci F, Marzano N, Soricelli A, Ferri R, Triggiani AI, Prestia A, Salinari S, Rasser PE, Basar E, Famà F, Nobili F, Yener G, Emek-Savaş DD, Gesualdo L, Mundi C, Thompson PM, Rossini PM, Frisoni GB (2015). Occipital sources of resting-state alpha rhythms are related to local gray matter density in subjects with amnesic mild cognitive impairment and Alzheimer’s disease. Neurobiol Aging.

[CR9] Baillet S, Mosher JC, Leahy RM (2001). Electromagnetic brain mapping. IEEE Signal Process Mag.

[CR10] Barnett L, Seth AK (2014). The MVGC multivariate Granger causality toolbox: a new approach to Granger-causal inference. J Neurosci Methods.

[CR11] Blinowska KJ (2011). Review of the methods of determination of directed connectivity from multichannel data. Med Biol Eng Comput.

[CR12] Blinowska KJ, Zygierewicz J (2011). Practical biomedical signal analysis using MATLAB. Series in medical physics and biomedical engineering.

[CR13] Boccaletti S, Latora V, Moreno Y, Chavez M, Hwanga DU (2006). Complex networks: structure and dynamics. Phys Rep.

[CR14] Braak H, Thal DR, Ghebremedhin E, Del Tredici K (2011). Stages of the pathologic process in Alzheimer disease: age categories from 1 to 100 years. J Neuropathol Exp Neurol.

[CR15] Bressler SL, Seth AK (2011). Wiener–Granger causality: a well established methodology. NeuroImage.

[CR16] Brier MR, Thomas JB, Fagan AM, Hassenstab J, Holtzman DM, Benzinger TL, Morris JC, Ances BM (2014). Functional connectivity and graph theory in preclinical Alzheimer’s disease. Neurobiol Aging.

[CR17] Brunner C, Billinger M, Seeber M, Mullen TR, Makeig S (2016). Volume conduction influences scalp-based connectivity estimates. Front Comput Neurosci.

[CR18] Burle B, Spieser L, Roger C, Casini L, Hasbroucq T, Vidal F (2015). Spatial and temporal resolutions of EEG: is it really black and white? A scalp current density view. Int J Psychophysiol.

[CR19] Canuet L, Tellado I, Couceiro V, Fraile C, Fernandez-Novoa L, Ishii R, Takeda M, Cacabelos R (2012). Resting-state network disruption and APOE genotype in Alzheimer’s disease: a lagged functional connectivity study. PLoS ONE.

[CR20] Chella F, Pizzella V, Zappasodi F, Marzetti L (2016). Impact of the reference choice on scalp EEG connectivity estimation. J Neural Eng.

[CR21] Cui J, Xu L, Bressler SL, Ding M, Liang H (2008). BSMART: a MATLAB/C toolbox for analysis of multichannel neural time series. Neural Netw.

[CR22] Dauwels J, Vialatte F, Musha T, Cichocki A (2010). A comparative study of synchrony measures for the early diagnosis of Alzheimer’s disease based on EEG. NeuroImage.

[CR23] Dauwels J, Vialatte F, Cichocki A (2010). Diagnosis of Alzheimer’s disease from EEG signals: where are we standing? [Review]. Curr Alzheimer Res.

[CR24] Davatzikos C, Bhatt P, Shaw LM, Batmanghelich KN, Trojanowski JQ (2011). Prediction of MCI to AD conversion, via MRI, CSF biomarkers, and pattern classification. Neurobiol Aging.

[CR25] de Haan W, Pijnenburg YA, Strijers RL, van der Made Y, van der Flier WM, Scheltens P, Stam CJ (2009). Functional neural network analysis in frontotemporal dementia and Alzheimer’s disease using EEG and graph theory. BMC Neurosci.

[CR26] Delbeuck X, Van der Linden M, Collette F (2003). Alzheimer’s disease as a disconnection syndrome? [Review]. Neuropsychol Rev.

[CR27] Ding M, Bressler S, Yang W, Liang H (2000). Short-window spectral analysis of cortical event-related potentials by adaptive multivariate autoregressive modeling: data preprocessing, model validation, and variability assessment. Biol Cybern.

[CR28] Dong L, Li F, Liu Q, Wen X, Lai Y, Xu P, Yao D (2017). MATLAB toolboxes for reference electrode standardization technique (REST) of scalp EEG. Front Neurosci.

[CR29] Dubois B, Feldman HH, Jacova C, Hampel H, Molinuevo JL, Blennow K, DeKosky ST, Gauthier S, Selkoe D, Bateman R, Cappa S, Crutch S, Engelborghs S, Frisoni GB, Fox NC, Galasko D, Habert MO, Jicha GA, Nordberg A, Pasquier F, Rabinovici G, Robert P, Rowe C, Salloway S, Sarazin M, Epelbaum S, de Souza LC, Vellas B, Visser PJ, Schneider L, Stern Y, Scheltens P, Cummings JL (2014). Advancing research diagnostic criteria for Alzheimer’s disease: the IWG-2 criteria. Lancet Neurol.

[CR30] Falasca NW, D’Ascenzo S, Di Domenico A, Onofrj M, Tommasi L, Laeng B, Franciotti R (2015). Hemispheric lateralization in top-down attention during spatial relation processing: a Granger causal model approach. Eur J Neurosci.

[CR31] Fisch BJ, Schomerand DL, daSilva FHL (2012). Polarity and field determinations. Niedermeyer’s electroencephalography: basic principles, clinical applications, and related fields.

[CR32] Florin E, Gross J, Pfeifer J, Fink GR, Timmermann L (2010). The effect of filtering on Granger causality based multivariate causality measures. NeuroImage.

[CR33] Franciotti R, Iacono D, Della Penna S, Pizzella V, Torquati K, Onofrj M, Romani GL (2006). Cortical rhythms reactivity in AD, LBD and normal subjects: a quantitative MEG study. Neurobiol Aging.

[CR34] Franciotti R, Falasca NW, Bonanni L, Anzellotti F, Maruotti V, Comani S, Thomas A, Tartaro A, Taylor JP, Onofrj M (2013). Default network is not hypoactive in dementia with fluctuating cognition: an Alzheimer disease/dementia with Lewy bodies comparison. Neurobiol Aging.

[CR35] Friston KJ (2011). Functional and effective connectivity: a review [Review]. Brain Connect.

[CR36] Geweke J (1982). Measurement of linear dependence and feedback between multiple time series. J Am Stat Assoc.

[CR37] Granger CW (1969). Investigating causal relations by econometric models and cross-spectral methods. Econ J Econ Soc.

[CR38] Guevara R, Velazguez JLP, Nenadovic V, Wennberg R, Senjanovic G, Dominguez LG (2005). Phase synchronization measurements using electroencephalographic recordings. What can we really say about neuronal synchrony?. Neuroinformatics.

[CR39] Haufe S, Nikulin V, Nolte G (2011). Identifying brain effective connectivity patterns from EEG: performance of Granger Causality, DTF, PDC and PSI on simulated data. BMC Neurosci.

[CR40] He Y, Chen Z, Evans A (2008). Structural insights into aberrant topological patterns of large-scale cortical networks in Alzheimer’s disease. J Neurosci.

[CR41] Hillebrand A, Tewarie P, van Dellen E, Yu M, Carbo EWS, Douw L, Gou AA, van Straaten EC, Stam CJ (2016). Direction of information flow in large-scale resting-state networks is frequency-dependent. Proc Natl Acad Sci USA.

[CR42] Huang Y, Zhang J, Cui Y, Yang G, He L, Liu Q, Yin G (2017). How different EEG references influence sensor level functional connectivity graphs. Front Neurosci.

[CR43] Jelles B, Scheltens P, van der Flier WM, Jonkman EJ, da Silva FH, Stam CJ (2008). Global dynamical analysis of the EEG in Alzheimer’s disease: frequency-specific changes of functional interactions. Clin Neurophysiol.

[CR44] Jeong J (2004). EEG dynamics in patients with Alzheimer’s disease. Clin Neurophysiol.

[CR45] Kaminski M, Blinowska KJ (2014). Directed Transfer Function is not influenced by volume conduction-inexpedient pre-processing should be avoided. Front Comput Neurosci.

[CR46] Keller JN, Schmitt FA, Scheff SW, Ding Q, Chen Q, Butterfield DA, Markesbery WR (2005). Evidence of increased oxidative damage in subjects with mild cognitive impairment. Neurology.

[CR47] Khadem A, Hossein-Zadeh GA (2014). Quantification of the effects of volume conduction on the EEG/MEG connectivity estimates: an index of sensitivity to brain interactions. Physiol Meas.

[CR48] Knott V, Mohr E, Mahoney C, Ilivitsky V (2000). Electroencephalographic coherence in Alzheimer’s disease: comparisons with a control group and population norms. J Geriatr Psychiatry Neurol.

[CR49] Koenig T, Prichep L, Dierks T, Hubl D, Wahlund LO, John ER, Jelic V (2005). Decreased EEG synchronization in Alzheimer’s disease and mild cognitive impairment. Neurobiol Aging.

[CR50] Latora V, Marchiori M (2010). Efficient behavior of small-world networks. Phys Rev Lett.

[CR51] Lee SJ, Park MH, Park SS, Ahn JY, Heo JH (2015). Quantitative EEG and medial temporal lobe atrophy in Alzheimer’s dementia: preliminary study. Ann Indian Acad Neurol.

[CR52] Lo CY, Wang PN, Chou KH, Wang J, He Y, Lin CP (2010). Diffusion tensor tractography reveals abnormal topological organization in structural cortical networks in Alzheimer’s disease. J Neurosci.

[CR53] Locatelli T, Cursi M, Liberati D, Franceschi M, Comi G (1998). EEG coherence in Alzheimer’s disease. Electroencephalogr Clin Neurophysiol.

[CR54] McKhann GM, Knopman DS, Chertkow H, Hyman BT, Jack CR, Kawas CH, Klunk WE, Koroshetz WJ, Manly JJ, Mayeux R, Mohs RC, Morris JC, Rossor MN, Scheltens P, Carrillo MC, Thies B, Weintraub S, Phelps CH (2011). The diagnosis of dementia due to Alzheimer’s disease: recommendations from the National Institute on Aging-Alzheimer’s Association workgroups on diagnostic guidelines for Alzheimer’s disease. Alzheimers Dement.

[CR55] Miraglia F, Vecchio F, Bramanti P, Rossini PM (2016). EEG characteristics in “eyes-open” versus “eyes-closed” conditions: small-world network architecture in healthy aging and age-related brain degeneration. Clin Neurophysiol.

[CR56] Mistur R, Mosconi L, Santi SD, Guzman M, Li Y, Tsui W, de Leon MJ (2009). Current challenges for the early detection of Alzheimer’s disease: brain imaging and CSF studies. J Clin Neurol.

[CR57] Moon JY, Lee U, Blain-Moraes S, Mashour GA (2015). General relationship of global topology, local dynamics, and directionality in large-scale brain networks. PLoS Comput Biol.

[CR58] Morbelli S, Piccardo A, Villavecchia G, Dessi B, Brugnolo A, Piccini A, Caroli A, Frisoni G, Rodriguez G, Nobili F (2010). Mapping brain morphological and functional conversion patterns in amnestic MCI: a voxel-based MRI and FDG-PET study. Eur J Nucl Med Mol Imaging.

[CR59] Morbelli S, Drzezga A, Perneczky R, Frisoni GB, Caroli A, van Berckel BN, Ossenkoppele R, Guedj E, Didic M, Brugnolo A, Sambuceti G, Pagani M, Salmon E, Nobili F (2012). Resting metabolic connectivity in prodromal Alzheimer’s disease. A European Alzheimer Disease Consortium (EADC) project. Neurobiol Aging.

[CR60] Mosconi L (2013). Glucose metabolism in normal aging and Alzheimer’s disease: methodological and physiological considerations for PET studies. Clin Transl Imaging.

[CR61] Newman ME (2002). Assortative mixing in networks. Phys Rev Lett.

[CR62] Nunez PL, Srinivasan R, Westdorp AF, Wijesinghe RS, Tucker DM, Silberstein RB, Cadusch PJ (1997). EEG coherency I: statistics, reference electrode, volume conduction, Laplacians, cortical imaging, and interpretation at multiple scales. Electroencephalogr Clin Neurophysiol.

[CR63] Pavlopoulos GA, Secrier M, Moschopoulos CN, Soldatos TG, Kossida S, Aerts J, Schneider R, Bagos PG (2011). Using graph theory to analyze biological networks. BioData Min.

[CR64] Petersen RC, Negash S (2008). Mild cognitive impairment: an overview [Review]. CNS Spectr.

[CR65] Petersen RC, Roberts RO, Knopman DS, Boeve BF, Geda YE, Ivnik RJ, Smith GE, Jack CR (2009). Mild cognitive impairment: ten years later [Review]. Arch Neurol.

[CR66] Picco A, Polidori MC, Ferrara M, Cecchetti R, Arnaldi D, Baglioni M, Morbelli S, Bastiani P, Bossert I, Fiorucci G, Brugnolo A, Dottorini ME, Nobili F, Mecocci P (2014). Plasma antioxidants and brain glucose metabolism in elderly subjects with cognitive complaints. Eur J Nucl Med Mol Imaging.

[CR67] Pogarell O, Teipel SJ, Juckel G, Gootjes L, Möller T, Bürger K, Leinsinger G, Möller HJ, Hegerl U, Hampel H (2005). EEG coherence reflects regional corpus callosum area in Alzheimer’s disease. J Neurol Neurosurg Psychiatry.

[CR68] Price JL, McKeel DW, Buckles VD, Roe CM, Xiong C, Grundman M, Hansen LA, Petersen RC, Parisi JE, Dickson DW, Smith CD, Davis DG, Schmitt FA, Markesbery WR, Kaye J, Kurlan R, Hulette C, Kurland BF, Higdon R, Kukull W, Morris JC (2009). Neuropathology of nondemented aging: presumptive evidence for preclinical Alzheimer disease. Neurobiol Aging.

[CR69] Reitz C, Brayne C, Mayeux R (2011). Epidemiology of Alzheimer disease. Nat Rev Neurol.

[CR70] Risacher SL, Saykin AJ, West JD, Shen L, Firpi HA, McDonald BC, Alzheimer, ’, s Disease Neuroimaging Initiative (ADNI) (2009). Baseline MRI predictors of conversion from MCI to probable AD in the ADNI cohort. Curr Alzheimer Res.

[CR71] Rubinov M, Sporns O (2010). Complex network measures of brain connectivity: uses and interpretations. NeuroImage.

[CR72] Ruscio J (2008). Constructing confidence intervals for Spearman’s rank correlation with ordinal data: a simulation study comparing analytic and bootstrap methods. J Mod Appl Stat Methods.

[CR73] Rusinek H, Endo Y, De Santi S, Frid D, Tsui WH, Segal S, Convit A, de Leon MJ (2004). Atrophy rate in medial temporal lobe during progression of Alzheimer disease. Neurology.

[CR74] Sanz-Arigita EJ, Schoonheim MM, Damoiseaux JS, Rombouts SA, Maris E, Barkhof F, Scheltens P, Stam CJ (2010). Loss of ‘small-world’ networks in Alzheimer’s disease: graph analysis of FMRI resting-state functional connectivity. PLoS ONE.

[CR75] Seth AK (2010). A MATLAB toolbox for Granger causal connectivity analysis. J Neurosci Methods.

[CR76] Seth AK, Barrett AB, Barnett L (2015). Granger causality analysis in neuroscience and neuroimaging. J Neurosci.

[CR77] Song J, Davey C, Poulsen C, Luu P, Turovets S, Anderson E, Li K, Tucker D (2015). EEG source localization: sensor density and head surface coverage. J Neurosci Methods.

[CR78] Sporns O, Honey CJ, Kötter R (2007). Identification and classification of hubs in brain networks. PLoS ONE.

[CR79] Stam CJ (2014). Modern network science of neurological disorders [Review]. Nat Rev Neurosci.

[CR80] Stam CJ, Jones BF, Nolte G, Breakspear M, Scheltens P (2007). Small-world networks and functional connectivity in Alzheimer’s disease. Cereb Cortex.

[CR81] Stam CJ, deHaan W, Daffertshofer A, Jones BF, Manshanden I, vanvan CappellenWalsum AM, Montez T, Verbunt JP, de Munck JC, van Dijk BW, Berendse HW, Scheltens P (2009). Graph theoretical analysis of magnetoencephalographic functional connectivity in Alzheimer disease. Brain.

[CR82] Thal DR, Rüb U, Orantes M (2002). Phases of Ab-deposition in the human brain and its relevance for the development of AD. Neurology.

[CR83] Tijms BM, Wink AM, de Haan W, van der Flier WM, Stam CJ, Scheltens P, Barkhof F (2013). Alzheimer’s disease: connecting findings from graph theoretical studies of brain networks [Review]. Neurobiol Aging.

[CR84] Valladares-Neto DC, Buchsbaum MS, Evans WJ, Nguyen D, Nguyen P, Siegel BV, Stanley J, Starr A, Guich S, Rice D (1995). EEG delta, positron emission tomography, and memory deficit in Alzheimer’s disease. Neuropsychobiology.

[CR85] van Straaten EC, Scheltens P, Gouw AA, Stam CJ (2014). Eyes-closed task-free electroencephalography in clinical trials for Alzheimer’s disease: an emerging method based upon brain dynamics. Alzheimers Res Ther.

[CR86] Vinck M, Huurdeman L, Bosman CA, Fries P, Battaglia FP, Pennartz CM, Tiesinga PH (2015). How to detect the Granger-causal flow direction in the presence of additive noise?. NeuroImage.

[CR87] Watts DJ, Strogatz SH (1998). Collective dynamics of ‘small-world’ networks. Nature.

[CR88] Yao Z, Zhang Y, Lin L, Zhou Y, Xu C, Jiang T (2010). Abnormal cortical networks in mild cognitive impairment and Alzheimer’s disease. PLoS Comput Biol.

[CR89] Zhao X, Liu Y, Wang X, Liu B, Xi Q, Guo Q, Jiang H, Jiang T, Wang P (2012). Disrupted small-world brain networks in moderate Alzheimer’s disease: a resting-state FMRI study. PLoS ONE.

